# Supplementary material for: Integrated Analysis of Metabolome and Transcriptome Reveals the Effect of Burdock Fructooligosaccharide on the Quality of Chinese Cabbage (Brassica rapa L. ssp. Pekinensis)
Source: Int J Mol Sci. 2024 Oct 25;25(21):11459. doi: 10.3390/ijms252111459 (PMC11546758; doi:10.3390/ijms252111459)
Supplement: Supplementary file 1 [file ijms-25-11459-s001.zip › Supplementary Figure 1.pdf]

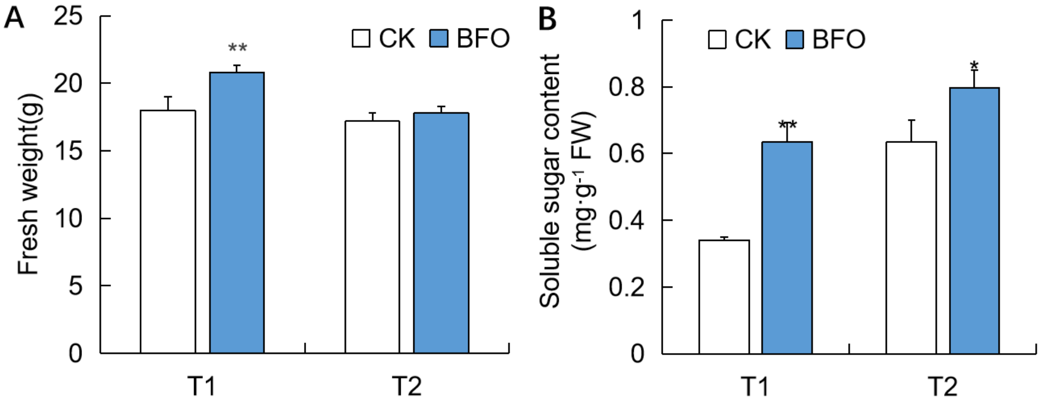

Supplementary Figure S1. Effect of BFO treatment on seedling the growth and quality of fast-growing vegetable. A, Effect of BFO treatment on fresh weight in fast-growing vegetable seedlings. B, Effect of BFO treatment on the soluble sugar content of fast-growing vegetable seedlings. T1 represents fast-vegetable variety '863', T2 represents fast-growing vegetable variety 'champion'. The Data are the means of three replicates (±SDs). Asterisks indicate statistical significance using Student' s t-test: *,P ⩽ 0.05, **,P ⩽ 0.01.
